# Bacteraemia with *Streptococcus agalactiae* – an observational study on clinical aspects and time to blood culture positivity

**DOI:** 10.1007/s10096-026-05411-w

**Published:** 2026-01-23

**Authors:** Torgny Sunnerhagen, Rima Mnajed, Alfred Törngren, Anna Bläckberg

**Affiliations:** 1 Division of Infection Medicine, Department of Clinical Sciences Lund, Lund University, Lund, Sweden; 2 Department of Clinical Microbiology, Skåne University Hospital Lund, Lund, Sweden; 3 Department of Infectious Diseases, Skåne University Hospital Lund, Lund, Sweden

**Keywords:** S. agalactiae, Time to blood culture positivity, Outcome, Infective endocarditis

## Abstract

**Purpose:**

*Streptococcus agalactiae* causes severe infections. Time to blood culture positivity (TTP) has been suggested as a marker of disease severity. This study investigated the clinical characteristics of *S. agalactiae* bacteraemia and the association between TTP and outcomes.

**Methods:**

This retrospective population-based study included adult episodes of *S. agalactiae* bacteraemia in southern Sweden, from 2016 to 2023. Medical records were reviewed. Information on TTP was obtained from the Department of Clinical Microbiology in Skåne. The primary outcome was 30-day mortality rate. Secondary outcomes were infective endocarditis (IE), development of sepsis and clinical deterioration within 48 h of blood culture collection.

**Results:**

A total of 463 patients were included. Median age was 72 years (interquartile range (IQR 62–82). Skin and soft tissue were the most common focus of infection. IE occurred in 23 patients. 30-day mortality was 9% (*n* = 40). TTP analysis included 411 patients. There was no statistically significant difference in levels of TTP between patients with 30-day mortality and survivors, 8.6 h (IQR 7.6–10.1) vs. 9.1 h (IQR 7.8–10.4), *p* = 0.4. Patients with IE had statistically significantly lower levels of TTP compared to patients without IE, 7.5 h (IQR 5.9–8.8) vs. 9.1 h (IQR 7.9–10.5), *p* = 0.005.

**Conclusion:**

*S. agalactiae* bacteraemia occurs in elderly patients, predominantly male, with a relatively high mortality. Shorter TTP was associated with the presence of IE, but not with mortality or sepsis, suggesting a potential role in identifying patients at risk for IE in *S. agalactiae* bacteraemia.

**Supplementary Information:**

The online version contains supplementary material available at 10.1007/s10096-026-05411-w.

## Introduction

*Streptococcus agalactiae*, a beta-haemolytic streptococcus commonly colonises the gastrointestinal and urogenital tracts, posing a risk of vertical-transmission-related sepsis, meningitis and pneumonia in newborns [1]. Beyond these neonatal complications, *S. agalactiae* is implicated in diverse diseases, ranging from skin and soft tissue infections, urinary tract infections, and infective endocarditis (IE) [2, 3].

Predicting adverse outcomes in patients with *S. agalactiae* infections poses a considerable challenge. Those with underlying comorbidities, such as diabetes and immunosuppression appear to be at an elevated risk of complications associated with *S. agalactiae* infections. Moreover, various laboratory parameters and imaging techniques have shown promise in anticipating and diagnosing severe outcomes linked to *S. agalactiae* infections, but identifying patients with *S. agalactiae* infections who are at high risk of developing a more unfavourable outcome is difficult.

Recent studies have emphasised the significance of time to blood culture positivity (TTP) as a prognostic factor in patients with bacteraemia caused by *Staphylococcus aureus*,* Klebsiella* spp. and *Streptococcus pneumonia*, and as a general predictor of adverse outcomes in bloodstream infections [4–7]. TTP, defined as the interval between blood culture bottle incubation and signal detection in an automated blood culture system, is postulated to mirror bacterial load [8].

Studies have found associations between TTP and an unfavourable outcome in bacteraemia with beta-haemolytic streptococci (BHS) in general (without determination of different species within the group) [9], and in more recent investigations involving *S. pyogenes and S. dysgalactiae*, encompassing the other groups of BHS, revealed that a shorter TTP was linked to a higher 30-day mortality rate [10, 11]. While TTP appears to be a valuable predictive tool in bacteraemia due to *S. pyogenes* and *S. dysgalactiae*, no studies have explored whether the same association holds for *S. agalactiae* bacteraemia.

*Streptococcus agalactiae* bacteraemia in adults has gained increasing attention in recent years. Although traditionally considered a pathogen associated with specific groups, reports from different regions indicate a rising incidence among the general adult population [12, 13]. Studies have described its clinical characteristics, prognostic factors, and outcomes, suggesting a notable disease burden, particularly among individuals with underlying comorbidities. Despite these findings, invasive *S. agalactiae* infections in adults remain relatively underexplored compared to other streptococcal species [14], highlighting the need for continued research into their epidemiology and clinical impact.

The present study aimed to investigate the characteristics of *S. agalactiae* bacteraemia in adults, and the possible association between levels of TTP and outcomes defined as primary outcome (30-day mortality rate) and secondary outcomes defined as the development of sepsis within 48 h from blood culture obtainment, the presence of IE and disease deterioration within 48 h from blood culture collection.

## Materials and methods

### Study cohort and setting

The study is a retrospective observational population-based study. Adult episodes of *S. agalactiae* bacteraemia that occurred between 2016 and 2023 were identified through the Department of Clinical Microbiology in Lund, Sweden. This Microbiology Clinic is responsible for microbiology diagnostics for all clinics (in- and outpatient) in Region Skåne, with a population of approximately 1.4 million in 2023. The five largest hospitals in the region (including the Skåne University Hospital in Lund and Malmö) were equipped with blood culture incubators where blood cultures could be placed at all times and were transported to the Clinical Microbiology laboratory when they turned positive. Five smaller hospitals lacked these blood culture incubators. These smaller hospitals instead sent their blood culture bottles directly to the Clinical Microbiology laboratory for incubation. The inclusion criteria were growth of *S. agalactiae* in blood culture. Exclusion criteria were patients under the age of 18 years, inaccessible medical records, and episodes with polymicrobial growth of the same blood culture bottle as *S. agalactiae*. In cases of recurrent bacteraemia, only the most recent episode of *S. agalactiae* bacteraemia was considered, as the outcome was mortality.

## Time to blood culture positivity

Information on levels of TTP was retrieved from the Department of Clinical Microbiology. If several blood cultures turned positive from the same patient, the shortest TTP was selected and included in the analysis. TTP was defined as the time between placement of the bottle in the incubator to the detection of growth, calculated automatically by the blood culture incubator software, and was retrieved from the laboratory information system. Information about the time between blood culture obtainment and the blood culture bottles being placed in the blood culture incubators was unavailable. The time to positivity was thus not adjusted for time to incubation.The blood culture system used in Region Skåne during the study period was BACTEC FX system (Becton Dickinson, Franklin Lakes, USA). Microflex MALDI-TOF MS (Bruker, Bremen, Germany) was used for species determination, using the MBT Compass Library version most updated at the time of analysis.

## Reviewing of medical records

Clinical characteristics of episodes of *S. agalactaie* bacteraemia were recorded including age, gender, place of acquisition (community, hospital or healthcare-related), the focus of infection, length of stay, Charlson comorbidity index (CCI) [15, 16] and antibiotic treatment. Health-care related infection was defined as a blood culture obtained in the initial 48 h from hospitalization if the patients had healthcare within 30 days prior to the episode, an outpatient or if the patient had been discharged from the hospital within 90 days prior to the current episode [17]. Focus of infection was defined as by fulfilment of at least two out of three different criteria based on signs of symptoms of an infection, microbiological evidence of *S. agalactiae* at the site of the infection, and imaging consistent with an infection. Erysipelas was an exception, as this is deemed a clinical diagnosis.

## Outcomes

Primary outcome for the TTP-analysis was defined as death within 30 days of blood culture obtainment. Secondary outcomes were defined as the development of sepsis within 6 and/or 48 h according to sepsis-3 criteria [18] from blood culture obtainment. The worst SOFA-scores for two different time intervals, 6 and 48 h from blood culture obtainment, were selected in order to detect any disease deterioration. IE was classified according to the Duke-ISCVID criteria [19].

## Statistics

For continuous variables, Mann-Whitney *U* was used. TTP was reported as median with interquartile ranges. Chi-square test was used for categorical data. Statistical analysis was performed in GraphPad Prism (GraphPad Software).

## Results

### Inclusion and exclusion of patients with S. agalactiae bacteraemia

**Fig. 1:**
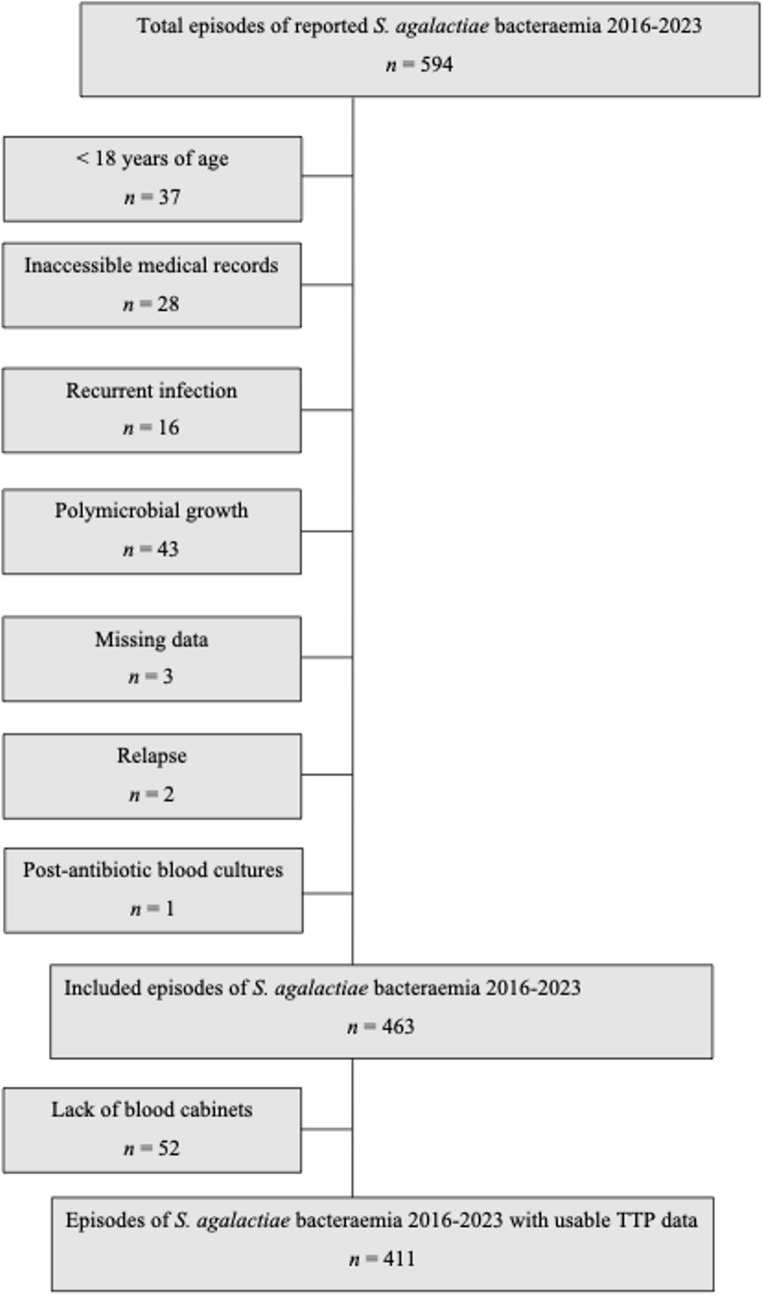
Flowchart of inclusion and exclusion of episodes of *Streptococcus agalactiae* bacteraemia

Between 2016 and 2023, a total of 170 571 individuals underwent at least one blood culture, of whom 38 877 had a positive result. During the same period, 594 adult episodes of *S. agalactiae* bacteraemia were identified. Of these 131 episodes were excluded for the following reasons: patient age < 18 years, inaccessible medical records (*n* = 28), recurrent infection (*n* = 16), polymicrobial growth (*n* = 43), missing data (*n* = 3), relapse (*n* = 2), and blood cultures drawn after initiation of antibiotic therapy (*n* = 1). This resulted in a final cohort of 463 individuals with *S. agalactiae* bacteraemia, representing 1.2% of all individuals with a positive blood culture during the study period. For the analysis of TTP, patients from hospitals without blood culture incubators were excluded (*n* = 52). Figure 1 summarises the inclusion and exclusion of episodes of *S. agalactiae* bacteraemia.

## Clinical characteristics

Table 1 summarises the clinical features of the whole study cohort of patients with *S. agalactiae* bacteraemia, as well as a comparison of patients and blood culture data from hospitals with and without blood culture incubators. Median age was 72 years (interquartile range (IQR 62–82), and the majority were male (56%). Skin and soft tissue were the most common focus of infection (*n* = 169), and 23 patients were diagnosed with IE. The 30-day mortality rate was 9% (*n* = 40).

**Table 1:** Patients with S. agalactiae bacteraemia and clinical characteristics

Clinical features	Total	Hospitals with incubators	Hospitals without incubators	*p*-value (with vs without incubators)
Age, median, (IQR), y	72 (62–82)	72 (62–82)	75 (68–83)	0.2
Sex, male, *n*(%),	56 (260)	233 (57)	27 (52)	0.6
CCI^a^, median (IQR)	1 (0–2)	1 (0–2)	2 (0–3)	0.1
Mode of acquisition, *n*(%)				
Community acquired	259 (56)	235 (57)	24 (46)	0.1
Healthcare related	178 (38)	151 (37)	27 (52)	0.05
Nosocomial	26 (6)	25 (6)	1 (2)	0.3
Focus of infection, *n*(%)				
Skin and soft tissue	169 (37)	151 (37)	18 (35)	0.9
Skeletal and joint	48 (10)	46 (11)	2 (4)	0.1
Respiratory tract	32 (7)	25 (6)	7 (13)	0.1
Postpartum infection	7 (2)	6 (1)	0 (0)	1.0
Infective endocarditis	23 (5)	18 (4)	5 (10)	0.2
Genitourinary tract	36 (8)	36 (9)	0 (0)	0.03
Unknown	155 (33)	135 (33)	20 (38)	0.06
Gastrointestinal	8 (2)	8 (2)	0 (0)	1.0
Postoperative infection	6 (1)	5 (1)	1 (2)	0.5
Other^b^	9 (2)	7 (2)	0 (0)	1.0
Adequate antibiotic treatment within 12 hours *n*(%)	410 (89)	366 (89)	44 (85)	0.4
Antibiotic treatment				
Length of treatment (intravenous), within 12 hours *n*(%)	6 (4–10)	6 (4–10)	6 (5–10)	0.2
Length of treatment (oral), median, (IQR), d	8 (5–10)	8 (5–10)	8 (6–10)	0.8
Length of treatment (total), median, (IQR), d	14 (11-17)	13 (11–17)	15 (12–19)	0.04
Patterns of antibiotic resistance, *n*(%)				
Amoxicillin	0 (0)	0 (0)	0 (0)	1.0
Penicillin G	0 (0)	0 (0)	0 (0)	1.0
Erythromycin	49 (11)	45 (11)	4 (8)	0.6
Clindamycin	50 (11)	45 (11)	5 (10)	1.0
Surgery, *n*(%)	45 (10)	42 (10)	3 (6)	0.5
Length of stay, median, (IQR), d	8 (5–12)	7,5 (5–12)	9 (6–13)	0.1
TTP, median, (IQR) h	8.8 (7.5–10.2)	9.1 (7.7–10.4)	6.6 (3.9–8.3)	< 0.0001
ICU, *n*(%)	16 (3)	14 (3)	2 (4)	0.7
Symptom duration before admission, median (IQR), d	1 (0–2)	1 (0–2)	1 (0–2)	0.6
Outcomes				
Mortality, 30 days, *n*(%)	40 (9)	40 (10)	0 (0)	0.01
Time to death, median, (IQR), d	7 (3-11)	7 (3-11)		
Mortality, 90 days, *n*(%)	53 (11)	51 (12)	2 (4)	0.1
Time to death, median, (IQR), d	10 (4-29)	9 (4-27)	43 (42-43)	0.1
Sepsis (0-48 h), *n*(%)	205 (44)	184 (45)	22 (42)	0.8
Septic shock (0-48 h), *n*(%)	20 (4)	18 (4)	2 (4)	1.0
Disease deterioration (0-6 to 6-48 h), *n*(%)	60 (13)	55 (13)	5 (10)	0.7

CCI: Charlson comorbidity index. TTP: time to positivity. ICU: intensive care unit.

^a^Points for age were not included in CCI. ^b^Other refers to tooth infection (*n* =1), mycotic aneurysm (*n* =1), mediastinitis (*n* =1), mastitis (n =2), and prosthesis infection (*n* =2)

### Primary outcome and secondary outcomes

**Fig. 2:**
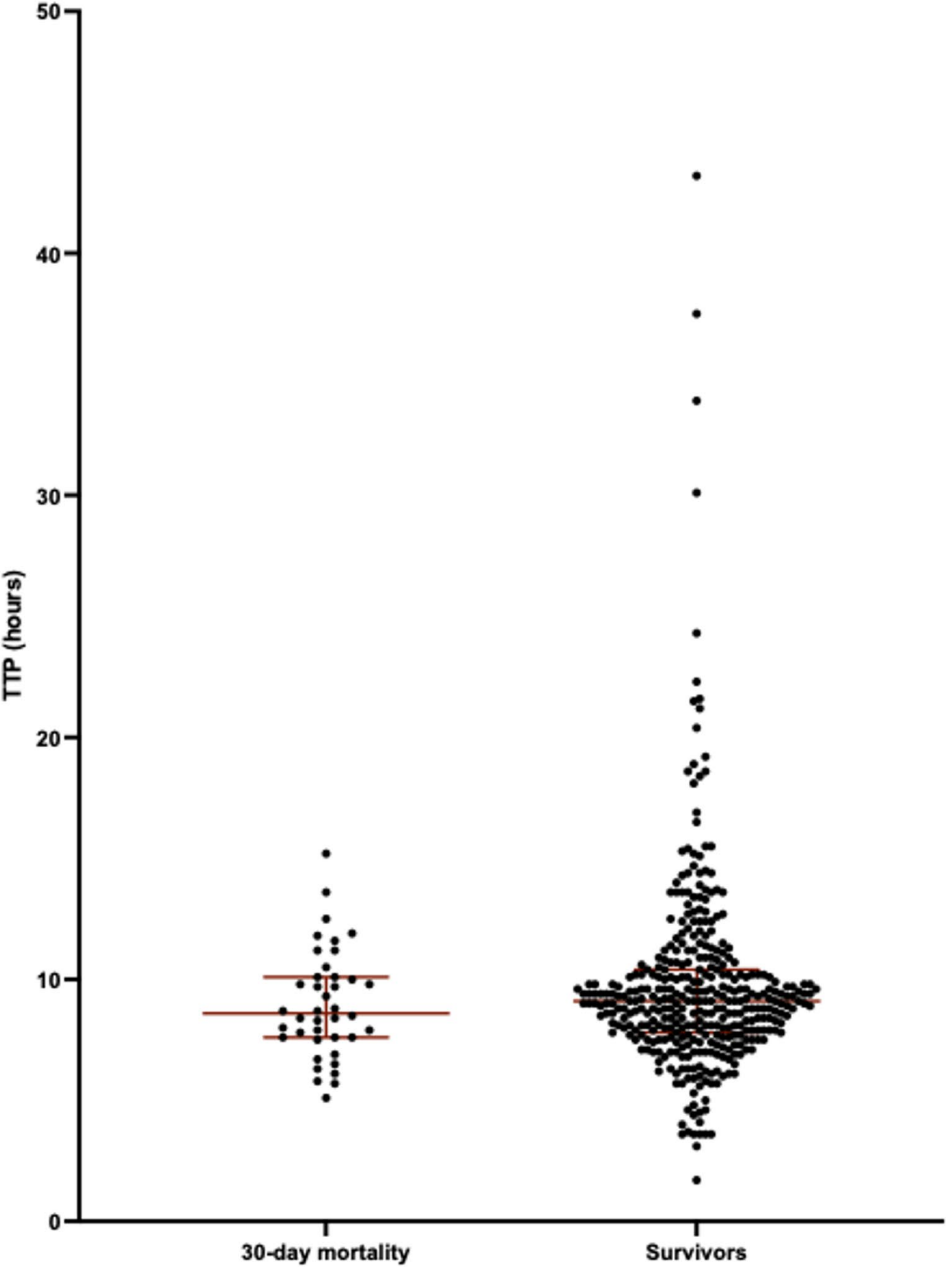
Survivors vs. non-survivors within 30 days after positive blood culture. Median TTP was shorter in patients who died within 30 days from blood culturing compared to survivors, but this was not statistically significant, 8.6 h (IQR 7.6–10.1) vs. 9.1 h (IQR 7.8–10.4), *p* = 0.4, Mann-Whitney *U* test

**Fig. 3:**
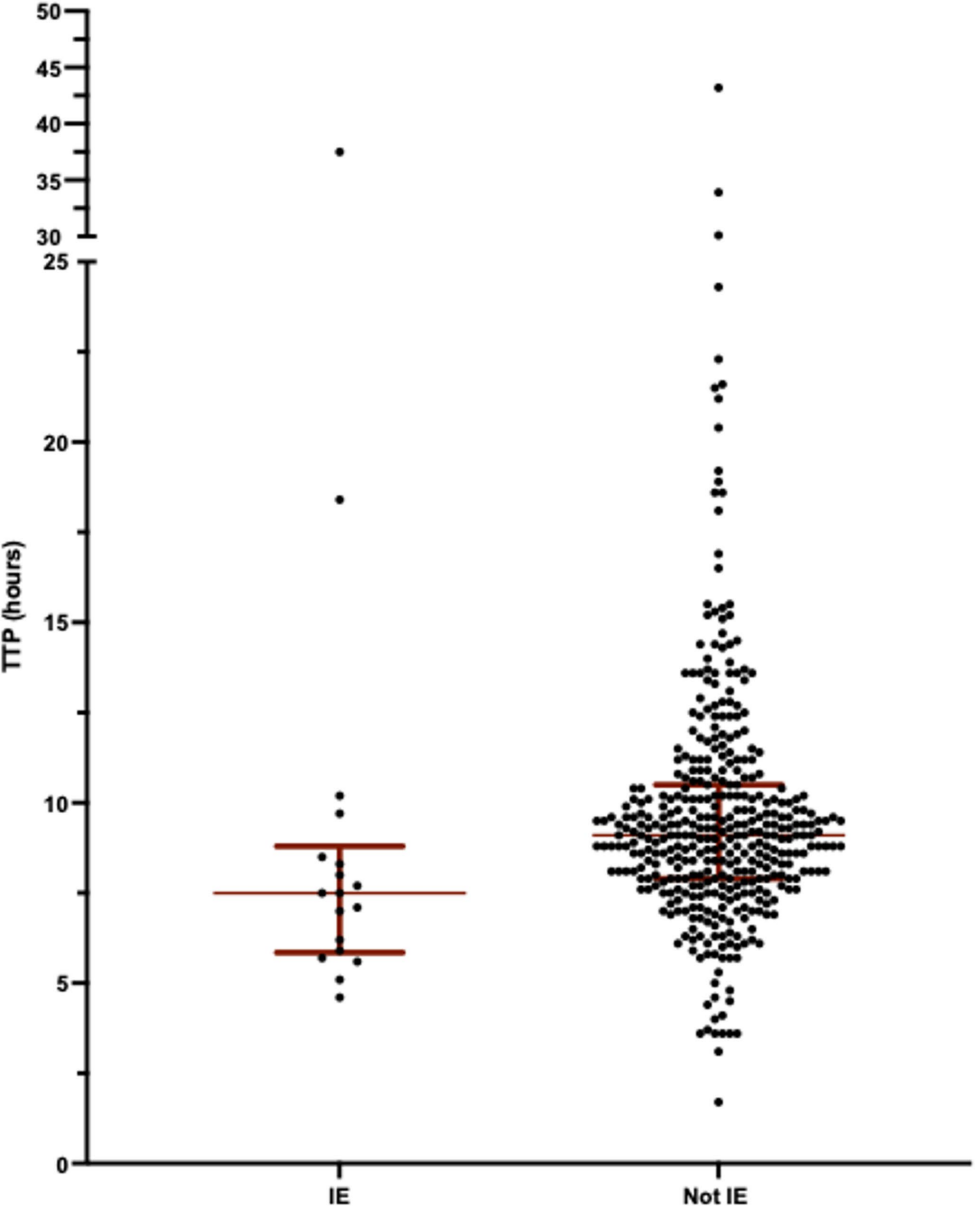
Time to positivity in patients with IE compared to patients with other foci of infection. Median TTP was statistically significantly shorter in patients with IE compared to patients without IE, 7.5 h (IQR 5.9–8.8) vs. 9.1 h (IQR 7.9–10.5), *p* = 0.005, Mann-Whitney *U* test

Table 2 summarises the analysis of outcome and association with TTP. Median TTP was shorter in patients who died within 30 days from blood culturing compared to survivors, but this was not statistically significant, 8.6 h (IQR 7.6–10.1) vs. 9.1 (IQR 7.8–10.4), *p* = 0.4, Mann-Whitney *U* test, Fig. 2. Median TTP was shorter in patients with IE compared to patients without IE, 7.5 h (IQR 5.9–8.8) vs. 9.1 (IQR 7.9–10.5), *p* = 0.005, Mann-Whitney *U* test, Fig. 3. There was no statistical difference in levels of TTP and development of sepsis and or any disease deterioration.

**Table 2:** Secondary outcomes and association with levels of TTP

Variable		*p* value
Sepsis (*n*=184)	Non-sepsis (*n* =227)	*p* = 0.3
Median TTP 8.8 hours (IQR 7.5-10.8)	Median 9.1 hours (IQR 8.0-10.2)	
90-day mortality (*n* = 51)	Survivors (*n* =359)	*p* = 0.4
Median 8.8 hours (IQR 7.6-10.1)	Median 9.1 hours (IQR 7.8-10.4)	
IE (*n* =18)	Non-IE (*n* =393)	*p* = 0.005
Median 7.5 hours (IQR 5.9-8.8)	Median 9.1 hours (IQR 7.9-10.5)	
Disease deterioration (*n* = 55)	No disease deterioration (*n* =356)	*p* = 0.2
Median 9.0 hours (IQR 7.3-9.8)	(Median 9.1 hours (IQR 7.8-10.5)	

TTP: time to blood culture positivity. IQR: interquartile range. IE: infective endocarditis

## Discussion

This cohort of 463 patients with *S. agalactiae* bacteraemia has a slightly higher median age than other studies [12–14]. The presence of multiple comorbidities and skin and soft tissue as a focus of infection is in line with previous studies, with the 30-day mortality of 9% somewhat lower than in previous studies [12, 13]. In this large population-based cohort, we found no significant association between TTP and 30-day mortality or early sepsis development in *S. agalactiae* bacteraemia. Notably, our findings deviate from two other published studies that encompassed different groups of beta-haemolytic streptococci [10, 11], in which IE is uncommon. Our cohort allowed assessment of the association between TTP and IE in *S. agalactiae* bacteraemia. We found that patients with IE had significantly shorter TTP values, suggesting that TTP may reflect a higher bacterial load and more invasive infection, and a result in line with what has been found for *Staphylococcus aureus* bacteraemia [4].

Beta-haemolytic streptococci grow at room temperature. This may explain the lower TTP values observed in blood cultures obtained from hospitals lacking blood culture incubators, where samples have to be transported to larger hospitals before incubation. In our dataset, TTP values were statistically significantly shorter in blood cultures originating from these hospitals compared with those from hospitals equipped with on-site incubators, Fig S.1 A major limitation of our study is the lack of precise data on the time intervals between blood culture sampling and incubation. This includes both transport times from hospitals without incubators to the central laboratory and the time from sampling to incubation within the five hospitals with incubators. Because TTP is highly dependent on the actual incubation start time, and because we could not determine this time point reliably for samples from hospitals without incubators, these patients had to be excluded from the analyses comparing TTP and clinical outcomes. Their TTP values are strongly influenced by pre-analytical delays, and therefore not comparable to those from hospitals where incubation begins immediately. The hospitals without incubators are also the smallest in the region, and lack intensive care units, intermediate care units, general surgery, obstetrics and night-time emergency services. This means that the patient population at these hospitals differ from that of larger hospitals, which may introduce a selection bias into comparisons of both TTP and clinical outcomes. Additional limitations include the retrospective study design and the absence of data on the volume of blood collected in each culture bottle, a factor known to affect TTP. Together, these limitations restrict our ability to fully assess how pre-analytical delays may have influenced the measurement of TTP or the clinical course of patients with *S. agalactiae* bacteraemia. Sepsis detection was constrained, considering we only considered two distinct time intervals, potentially overlooking patients who developed sepsis more than 48 h post blood culture obtainment. Nevertheless, the study´s strengths include its relatively large population-based cohort, design and comprehensive microbiological data, providing insights into the clinical significance of TTP in *S. agalactiae* bacteraemia.

## Conclusion

*S. agalactiae* bacteraemia primarily affects older adults with comorbidities, most commonly presenting with skin and soft tissue infections and carries a relatively high mortality rate. Shorter TTP was associated with IE, but not with mortality or sepsis in *S. agalactiae* bacteraemia. Further prospective studies are warranted to determine whether incorporating TTP into diagnostic algorithms could improve early identification of endocarditis and guide clinical management.

## Supplementary Information

Below is the link to the electronic supplementary material.

Supplementary Figure S1 Comparison of time to positivity in patients with blood cultures obtained from hospitals with and without blood culture cabinets. Median TTP was statistically significantly shorter in patients with blood cultures acquired from hospitals without blood culture cabinets compared to blood cultures obtained from hospitals with culture cabinets, 6.6 hours (IQR 3.9-8.3) vs 9.1 hours (IQR 7.7-10.4), *p* < 0.0001, Mann-Whitney U test.(PNG 224 KB)

Supplementary Material 1

## Data Availability

Due to the terms of the ethical review permit, no data that can be linked to individual patients can be shared.

## References

[1] Seale AC, Bianchi-Jassir F, Russell NJ, Kohli-Lynch M, Tann CJ, Hall J et al (2017) Estimates of the burden of group B Streptococcal disease worldwide for pregnant Women, Stillbirths, and children. Clin Infect Dis 65(suppl2):S200–s19 29117332 10.1093/cid/cix664 PMC5849940

[2] Farley MM (2001) Group B Streptococcal disease in nonpregnant adults. Clin Infect Dis 33(4):556–561 11462195 10.1086/322696

[3] Oravec T, Oravec SA, Leigh J, Matthews L, Ghadaki B, Mertz D et al (2022) Streptococcus agalactiae infective endocarditis in canada: a multicenter retrospective nested case control analysis. BMC Infect Dis 22(1):18 34983419 10.1186/s12879-021-06997-6 PMC8725325

[4] Siméon S, Le Moing V, Tubiana S, Duval X, Fournier D, Lavigne JP et al (2019) Time to blood culture positivity: an independent predictor of infective endocarditis and mortality in patients with Staphylococcus aureus bacteraemia. Clin Microbiol Infect 25(4):481–488 30036664 10.1016/j.cmi.2018.07.015

[5] Hou W, Han T, Qu G, Sun Y, Yang D, Lin Y (2023) Is early time to positivity of blood culture associated with clinical prognosis in patients with Klebsiella pneumoniae bloodstream infection? Epidemiol Infect 151:e43 36805070 10.1017/S0950268823000262 PMC10028975

[6] Cillóniz C, Ceccato A, de la Calle C, Gabarrús A, Garcia-Vidal C, Almela M et al (2017) Time to blood culture positivity as a predictor of clinical outcomes and severity in adults with bacteremic Pneumococcal pneumonia. PLoS ONE 12(8):e0182436 28787020 10.1371/journal.pone.0182436 PMC5546626

[7] Ljungquist O, Tverring J, Oldberg K, Sunnerhagen T, Torisson G (2025) Association of time to positivity with disease severity in bloodstream infections-a population-based cohort study. Clin Microbiol Infect 31(9):1532–1538 40447220 10.1016/j.cmi.2025.05.027

[8] Lamy B (2019) Blood culture time-to-positivity: making use of the hidden information. Clin Microbiol Infect 25(3):268–271 30580034 10.1016/j.cmi.2018.12.001

[9] Hamilton F, Evans R, Ghazal P, MacGowan A (2022) Time to positivity in bloodstream infection is not a prognostic marker for mortality: analysis of a prospective multicentre randomized control trial. Clin Microbiol Infect 28(1):136e7–e13 10.1016/j.cmi.2021.05.043 34111588

[10] Bläckberg A, Svedevall S, Lundberg K, Nilson B, Kahn F, Rasmussen M (2022) Time to blood culture positivity: an independent predictor of mortality in Streptococcus pyogenes bacteremia. Open Forum Infect Dis 9(6):ofac163 35615297 10.1093/ofid/ofac163 PMC9126491

[11] Bläckberg A, Lundberg K, Svedevall S, Nilson B, Rasmussen M (2023) Time to positivity of blood cultures in bloodstream infections with Streptococcus dysgalactiae and association with outcome. Infect Dis (Lond) 55(5):333–339 36847483 10.1080/23744235.2023.2182910

[12] Collin SM, Shetty N, Lamagni T (2020) Invasive group B Streptococcus infections in Adults, England, 2015–2016. Emerg Infect Dis 26(6):1174–1181 32441619 10.3201/eid2606.191141 PMC7258460

[13] Vasikasin V, Changpradub D (2021) Clinical manifestations and prognostic factors for Streptococcus agalactiae bacteremia among nonpregnant adults in Thailand. J Infect Chemother 27(7):967–971 33610481 10.1016/j.jiac.2021.02.010

[14] Park JH, Jung J, Kim MJ, Sung H, Kim MN, Chong YP et al (2019) Incidence, clinical characteristics, and outcomes of Streptococcus dysgalactiae subspecies equisimilis bacteremia in a tertiary hospital: comparison with S. agalactiae bacteremia. Eur J Clin Microbiol Infect Dis 38(12):2253–2258 31392445 10.1007/s10096-019-03667-z

[15] Charlson ME, Pompei P, Ales KL, MacKenzie CR (1987) A new method of classifying prognostic comorbidity in longitudinal studies: development and validation. J Chronic Dis 40(5):373–383 3558716 10.1016/0021-9681(87)90171-8

[16] Quan H, Li B, Couris CM, Fushimi K, Graham P, Hider P et al (2011) Updating and validating the Charlson comorbidity index and score for risk adjustment in hospital discharge abstracts using data from 6 countries. Am J Epidemiol 173(6):676–682 21330339 10.1093/aje/kwq433

[17] Friedman ND, Kaye KS, Stout JE, McGarry SA, Trivette SL, Briggs JP et al (2002) Health care–associated bloodstream infections in adults: a reason to change the accepted definition of community-acquired infections. Ann Intern Med 137(10):791–797 12435215 10.7326/0003-4819-137-10-200211190-00007

[18] Singer M, Deutschman CS, Seymour CW, Shankar-Hari M, Annane D, Bauer M et al (2016) The third international consensus definitions for sepsis and septic shock (Sepsis-3). JAMA 315(8):801–810 26903338 10.1001/jama.2016.0287 PMC4968574

[19] Fowler VG, Durack DT, Selton-Suty C, Athan E, Bayer AS, Chamis AL et al (2023) The 2023 Duke-International society for cardiovascular infectious diseases criteria for infective endocarditis: updating the modified Duke criteria. Clin Infect Dis 77(4):518–526 37138445 10.1093/cid/ciad271 PMC10681650

