# Supplementary figures and images for: Bacteraemia with Streptococcus agalactiae – an observational study on clinical aspects and time to blood culture positivity
Source: Eur J Clin Microbiol Infect Dis. 2026 Jan 23;45(5):1275–83. doi: 10.1007/s10096-026-05411-w (PMC13222174; doi:10.1007/s10096-026-05411-w)

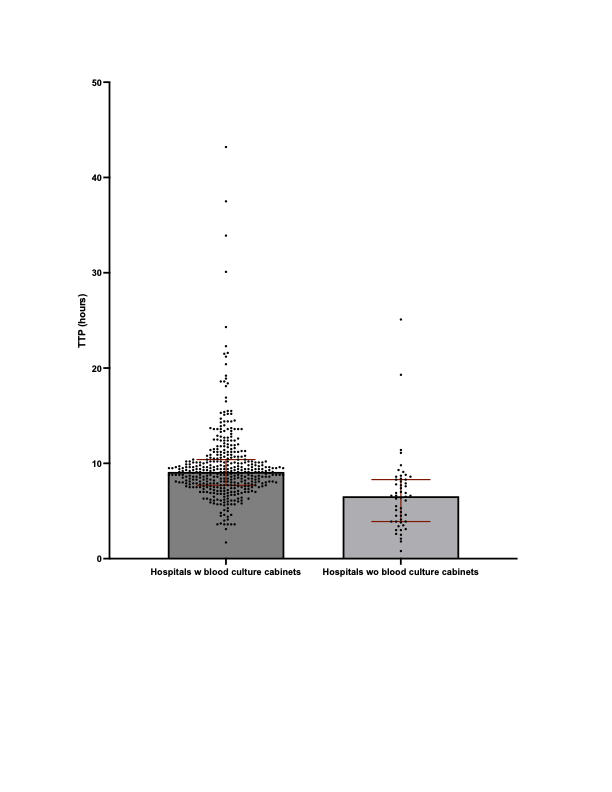

Supplement: Supplementary file 1 — Comparison of time to positivity in patients with blood cultures obtained from hospitals with and without blood culture cabinets. Median TTP was statistically significantly shorter in patients with blood cultures acquired from hospitals without blood culture cabinets compared to blood cultures obtained from hospitals with culture cabinets, 6.6 hours (IQR 3.9-8.3) vs 9.1 hours (IQR 7.7-10.4), p < 0.0001, Mann-Whitney U test.(PNG 224 KB) [file 10096_2026_5411_Fig4_ESM.png]

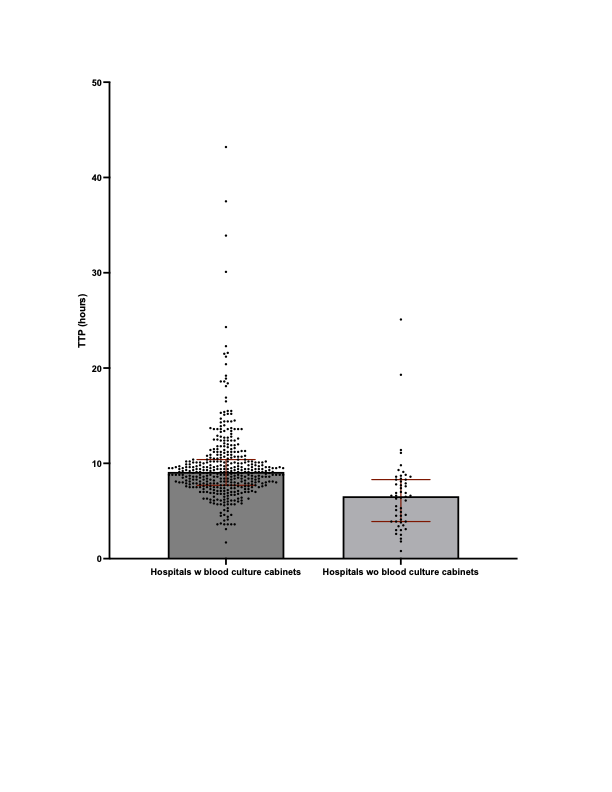

Supplement: Supplementary file 2 — Supplementary Material 1 [file 10096_2026_5411_MOESM1_ESM.tiff]
